# Overcoming Intrapulmonary Shunting: A Case Report of Hepatopulmonary Syndrome Post‐Liver Transplantation Treated With Inhaled Treprostinil

**DOI:** 10.1002/rcr2.70204

**Published:** 2025-05-19

**Authors:** John Fanous, Dakota McNierney, Abdelmohaymin Abdalla, Jehad Azar

**Affiliations:** ^1^ Department of Pulmonary and Critical Care Medicine Mayo Clinic Phoenix Arizona USA

**Keywords:** hepatopulmonary syndrome, intrapulmonary shunting, liver transplantation, Treprostinil

## Abstract

Hepatopulmonary syndrome (HPS) poses a significant challenge in liver transplant recipients, mostly resulting in persistent hypoxaemia postoperatively. We present the case of a 59‐year‐old male with decompensated metabolic dysfunction associated with steatohepatitis (MASH) cirrhosis, who had liver transplantation due to end‐stage liver disease complicated by HPS‐related hypoxaemia. Despite successful transplantation, the patient continued to experience severe hypoxaemia, requiring high supplemental oxygen despite maximal conservative treatment per guidelines. Potential causes for hypoxaemia post‐liver transplant have been excluded; HPS as the sole cause of hypoxaemia was proved with a repeat shunt study, which estimated the shunt at 59%. Treatment with inhaled Treprostinil led to a remarkable improvement in oxygenation, facilitating the successful weaning of oxygen supplementation. The patient's hypoxaemia improved to full recovery upon discharge. This case highlights the persistence of hypoxaemia post‐liver transplantation in HPS patients and underscores the potential role of inhaled Treprostinil as a novel therapeutic approach to address this complication.

## Graphical abstract

Hepatopulmonary syndrome (HPS) poses a significant challenge in liver transplant recipients, often resulting in persistent hypoxaemia postoperatively. This case highlights the persistence of hypoxaemia post‐liver transplantation in HPS patients and underscores the potential role of inhaled Treprostinil as a novel therapeutic approach to address this complication.

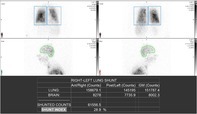

## 1 Introduction

Hepatopulmonary syndrome (HPS) presents a formidable challenge in patients with end‐stage liver disease, often complicating the management of liver transplantation. Despite its prevalence, therapeutic options for severe HPS‐related hypoxaemia post‐liver transplantation remain limited. We present the case of a 59‐year‐old male with decompensated metabolic dysfunction associated with steatohepatitis (MASH) cirrhosis, who continued to experience significant hypoxaemia post‐liver transplantation, necessitating high supplemental oxygen. However, treatment with inhaled Treprostinil proved to be a pivotal intervention, resulting in a notable improvement in oxygenation and facilitating successful weaning from high oxygen requirements. This case highlights the novel utility of inhaled Treprostinil in addressing severe hypoxaemia post‐liver transplantation in HPS patients.

## 2 Case Report

A 59‐year‐old male with a significant medical history of decompensated MASH‐associated cirrhosis, complicated by oesophageal variceal bleeding, ascites, severe malnutrition, hepatic hydrothorax and hepatic encephalopathy presented to our pulmonary clinic in August 2023 for pre‐liver transplant evaluation. He had previously been listed for a liver transplant at an outside facility but was removed from the waitlist due to severe hypoxaemia with a partial pressure of oxygen (pO_2_) < 45 mmHg on room air, which corrected to 257 mmHg on 100% fraction of inspired oxygen (FiO_2_). Pulmonary function testing was relatively normal besides a reduced DLCO of 20%. A previous shunt fraction assessment in 2022 revealed a value of 20%. Upon examination, the patient appeared comfortable, not in respiratory distress, and could speak in complete sentences while receiving 5 L of oxygen via nasal cannula. Chest auscultation revealed clear breath sounds bilaterally with no audible wheezes or rhonchi noted. The patient had been dependent on oxygen for the past 2.5 years with no prior history of lung disease, smoking, vaping or marijuana use. He reported exertional dyspnoea and was able to walk only about 10 ft before needing to pause to catch his breath. Arterial blood gas (ABG) demonstrated a pO_2_ of 34 mmHg at 21% FiO_2_, which increased to 377 mmHg at 100% FiO_2_. A stress echocardiogram showed normal left ventricular (LV) size with an ejection fraction (EF) of 66%, an estimated right ventricular systolic pressure of 22 mmHg, and a large intrapulmonary shunt with left heart filling bubbles noted after five cardiac cycles. A nuclear medicine shunt evaluation indicated the presence of a right‐to‐left shunt with a shunt index of 28.8%. Subsequently, from a pulmonary perspective, he was cleared to undergo liver transplantation, which took place in November 2023.

**FIGURE 1:**
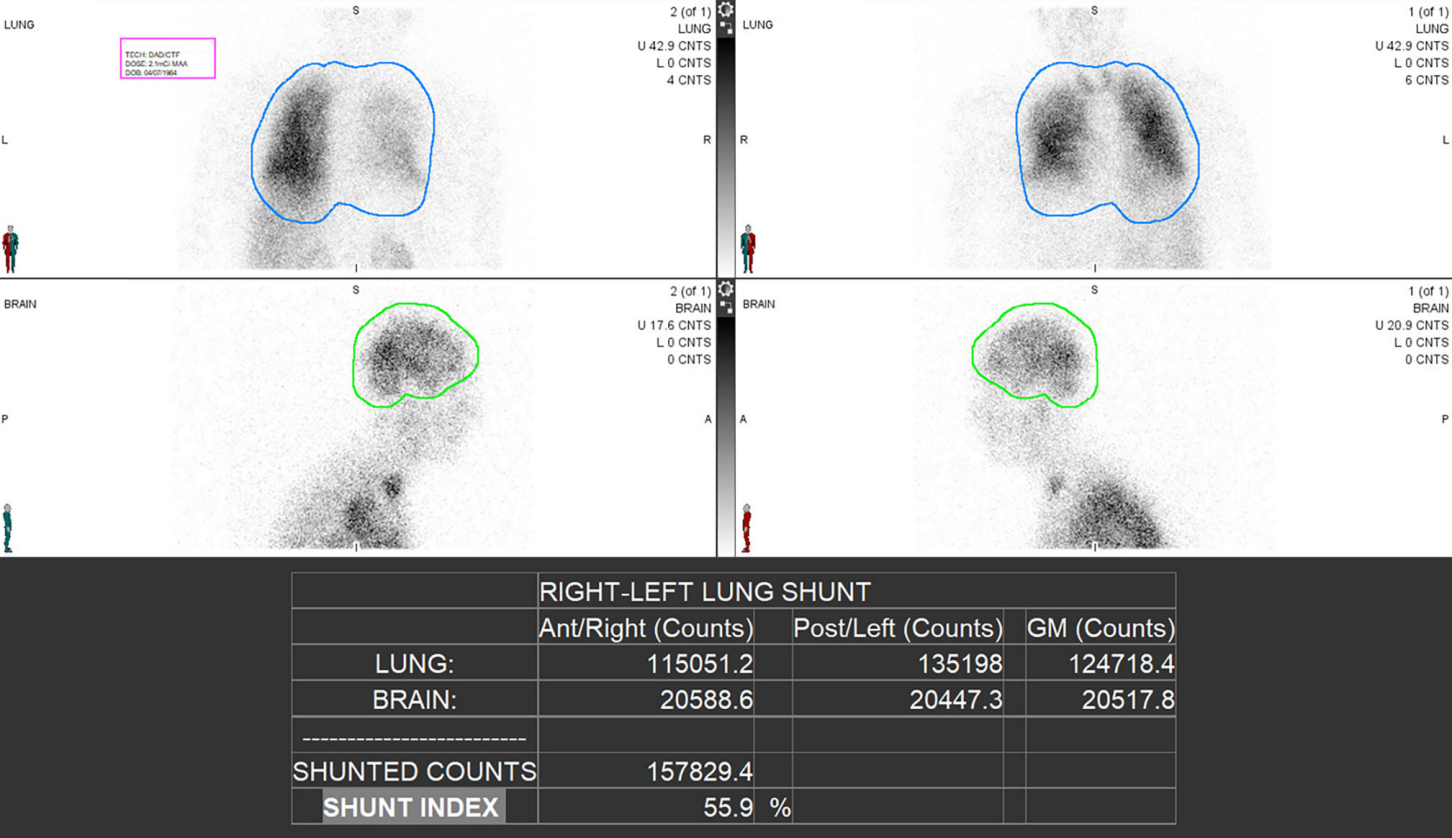
Nuclear medicine shunt evaluation post‐liver transplantation showing severe intrapulmonary shunting of 56%.

**FIGURE 2:**
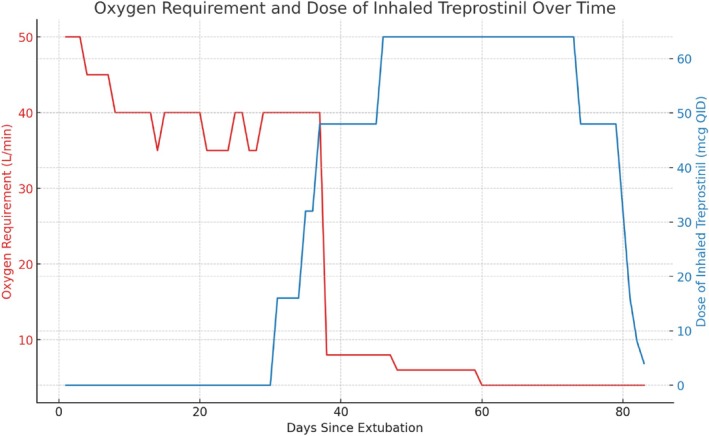
Oxygen requirement and dose of inhaled Treprostinil over time. The graph shows the patient's oxygen requirement (in L/min) decreasing as the dose of inhaled Treprostinil (in mcg QID) is titrated upwards.

**FIGURE 3:**
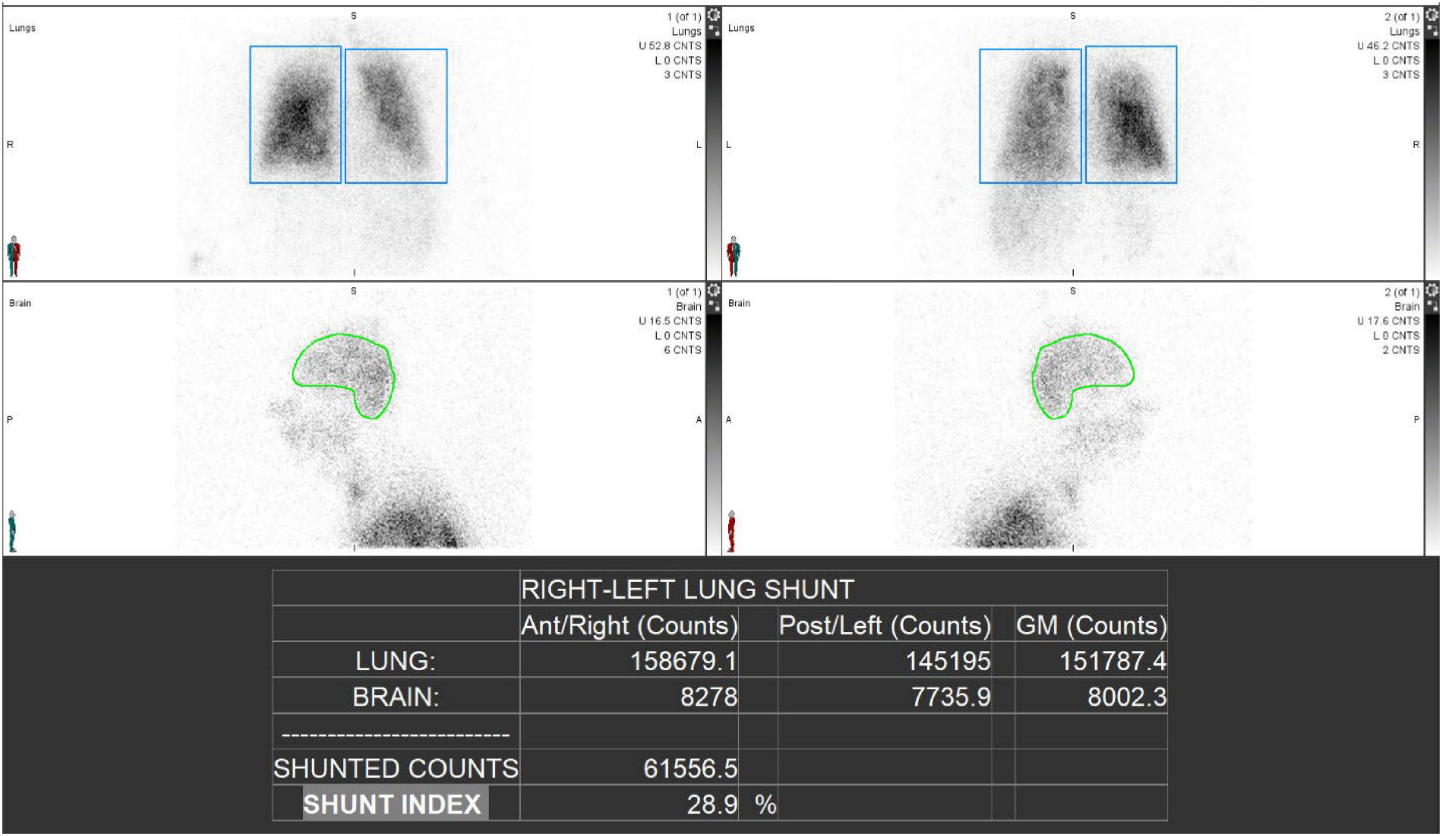
Nuclear medicine shunt evaluation post‐treatment with inhaled Treprostinil showing intrapulmonary shunting decreased to 29%.

Postoperatively, the patient was extubated to a high‐flow nasal cannula, delivering 0.70 FiO_2_ at 50 L/min. Given that his baseline oxygen requirement prior to liver transplantation was 6–8 L via nasal cannula with oxygen saturation around 90 mmHg, the pulmonary inpatient team was consulted for further evaluation and management. Pulmonary embolism was ruled out by CT angiography, with no signs of chronic thromboembolic pulmonary disease (CTEPD), and a negative deep vein thrombosis (DVT) scan. Repeat echocardiography revealed diastolic dysfunction, normal LV systolic function and no evidence of pulmonary hypertension. After the exclusion of other causes of hypoxaemia and the presence of severe intrapulmonary shunting that increased to 56% post‐transplant on repeat nuclear medicine shunt evaluation (Figure 1), the patient was initiated on inhaled Treprostinil for severe HPS post‐transplantation on Day 30 post‐extubation. Figure 2 shows the effect on oxygen requirements at different doses of Treprostinil: there was no significant improvement at 16 mcg QID (four times daily), a significant improvement at 32 mcg QID, a plateau at 48 mcg QID with minimal further effect noted at 64 mcg QID. The dose was titrated to 64 mcg QID without any significant side effects such as ventilation/perfusion mismatch or hypotension. He demonstrated significant clinical improvement, with enhanced functional capacity and a reduction in oxygen requirement to 5 L/min. Treprostinil was continued and eventually weaned off 52 days after starting it. At a follow‐up clinic appointment in March 2024, a repeat nuclear medicine shunt study showed improvement with a shunt index of 29% (Figure 3), and he was requiring 4–6 L of oxygen via nasal cannula.

## 3 Discussion

HPS is a known significant cause of hypoxia and dyspnoea in patients with end‐stage liver disease. It can be characterized by a triad of chronic liver disease, gas exchange abnormalities and the presence of intrapulmonary shunting [1]. Among cirrhotic patients, the prevalence ranges between 4% and 47%, and is often underdiagnosed due to coexisting comorbidities and pulmonary complications that obscure its diagnosis [2]. It is an escalating condition, with a decrease of 5 mmHg in PaO_2_ annually [3]. Without liver transplantation, HPS is associated with a more than twofold increase in mortality, which is why it remains the definitive treatment, as oxygenation typically improves post‐transplant. Notably, a pre‐transplant arterial oxygen pressure (PaO_2_) below 44 mmHg correlates with increased post‐transplant mortality, underscoring the necessity for timely intervention [4].

The pathophysiology of HPS is multifaceted as it involves increased nitric oxide (NO) production, monocyte‐driven inflammation and angiogenesis. Increased NO levels are caused by increased activity of endothelial nitric oxide synthase (eNOS) and inducible nitric oxide synthase (iNOS), which mediate pulmonary vasodilation, leading to ventilation‐perfusion (V/Q) mismatch [5]. Monocyte influx may be caused by bacterial translocation due to intestinal endotoxaemia, which aggravates this process through the production of proangiogenic factors such as vascular endothelial growth factor (VEGF) and platelet‐derived growth factor (PDGF) [6]. Angiogenesis further worsens HPS by increasing vascular permeability and shunting. Despite these insights, medical therapies targeting these pathways, including pentoxifylline and sorafenib, have shown limited clinical success.

Inhaled pulmonary vasodilators, such as Treprostinil, represent a novel therapeutic approach aimed at redistributing pulmonary blood flow to correct ventilation‐perfusion (V/Q) mismatch. Although this approach may seem counterintuitive, it leverages the concept that the most severely affected lung regions are already fully dilated, enabling targeted vasodilation in other areas to optimize perfusion. There have been a few case reports published highlighting its success [7, 8]. Similarly, methylene blue acts by inhibiting soluble guanylate cyclase (sGC) and counteracting NO‐mediated pathways, thereby reducing shunting and enhancing oxygenation [9]. Our patient showed significant improvement with his hypoxia, shunt index and quality of life within 3 months of Treprostinil initiation. This is at the lower end of lower compared to previous reports of resolution ranging from 2 to 14 months [10]. Although these therapies hold significant promise, their safety and efficacy require validation through large‐scale clinical trials. Additionally, interventional techniques like abnormal pulmonary vessel embolization offer potential benefits but are limited by procedural risks and the need for specialized expertise.

In conclusion, this case report highlights the significant challenges in managing severe, refractory hypoxaemia associated with HPS, even after liver transplantation. The use of inhaled Treprostinil resulted in a marked improvement in oxygenation and facilitated weaning from high supplemental oxygen, likely due to its effects on perfusion uniformity and reduction of ventilation–perfusion (V/Q) mismatch. These findings emphasize the potential role of inhaled pulmonary vasodilators as adjunctive therapy in HPS management. However, more extensive clinical trials are essential to confirm their safety, efficacy and broader applicability, paving the way for novel therapeutic strategies in this challenging condition.

## Author Contributions

All authors contributed to the writing and editing of this manuscript.

## Consent

The authors declare that appropriate written informed consent was obtained for the publication of this manuscript and accompanying images.

## Conflicts of Interest

The authors declare no conflicts of interest.

## Data Availability

Data sharing is not applicable to this article as no datasets were generated or analysed during the current study.
